# Comparisons between the circular restricted three-body and bi-circular four body problems for transfers between the two smaller primaries

**DOI:** 10.1038/s41598-022-08046-x

**Published:** 2022-03-09

**Authors:** Allan Kardec de Almeida Junior, Antonio Fernando Bertachini de Almeida Prado

**Affiliations:** 1 INPE: National Institute for Space Research, São José dos Campos, SP Brazil; 2 Peoples’ Friendship University of Russia (RUDN University), 6 Miklukho-Maklaya Street, 117198 Moscow, Russian Federation

**Keywords:** Planetary science, Space physics, Astronomy and planetary science, Engineering, Astronomy and astrophysics, Space physics

## Abstract

Important properties of the dynamics of a spacecraft can be obtained from the Circular Restricted Three Body Problem and the Bi-Circular Bi-planar Four Body Problem. In this work, both systems are compared under the perspective of the costs involved in a transfer between the smaller primaries. An analytical approach shows several properties of the perturbation due to the gravity of the Sun and the motion of the smaller primaries around it over a spacecraft in the region of interest, like its behavior at and around the barycenter or at any point in a circle around the Sun. The costs involved in transfers between the smaller primaries are numerically evaluated and analyzed using the newly developed Theory of Functional Connections. The results show that the influence of this perturbation over the costs is significant for systems like the Sun–Earth–Moon or Sun–Mars–Phobos. On the other hand, it is also shown that this influence may be negligible for other very different systems, like the Sun–Saturn–Titan or Sun–Ida–Dactyl. Maps of perturbation are drawn in the region of interest, which can be used for mission designers. Finally, a new approach to describe the influence of the Sun over the tides of the smaller primaries is proposed under the Four Body Problem model.

## Introduction

The three-body problem is described by Newton’s equations of motion for three bodies subjected to their mutual gravitational attraction. The circular restricted three-body problem (CR3BP) is a particular case where one of the bodies has a negligible mass (e.g. a satellite) and the motion of the other bodies are circular around their barycenter. Although there is no analytical closed (other than series) solution to this problem^1^, this model has been proven to be very useful in astrodynamics to find general features of orbits. This is possible due to its simplification from many perturbations to the few main ones in the region under consideration.

A combination of two constrained “three-body problems” was structured by^2^ to find qualitative properties of the motion of satellites under the gravitational influence of the Earth, Moon, and Sun. In general, the massive bodies in the first restricted three body problem are a planet (or an asteroid) and its moon, while the massive bodies in the second restricted three body problem are given by the Sun and the other two pairs combined. In a subsequent paper, mathematical descriptions of the very restricted four body problem was done by the same author in^3^. In comparison with the CR3BP, this model takes into consideration the gravitational influence of the Sun by adding a term which is a function of the position of the Sun relative to the Earth–Moon system.

The existence of periodic motion around the classical libration points of the CR3BP was shown for this four body problem (4BP) in^4^. Since then, the four body problem has been successfully investigated for specific masses and positions of the bodies in the space (under symmetrical configurations)^5–8^. Nevertheless, the restricted four body problem has attracted the interest of many researchers in astrodynamics, because it can be used to find properties of the motion of a satellite—its mass is much lower than the other three bodies. The restricted four body problem has also been investigated under specific configurations as the one in which all the primaries are located in a straight-line equilibrium configuration^9^ or all the primaries are located in the vertices of an equilateral triangle with equal^10^ or different masses^11^—a problem similar to the Hill’s approximation in which the mass of one of the primaries is much lower than the mass of the others. Furthermore, the conditions in which the bi-circular four body problem admits Jacobian integral or the energy conservation law are shown in^12^. The equilibria, stability and chaos in the bi-circular 4BP has also been recently investigated in^13^. This system has also been studied under dissipative forces^14^. A recent investigation shows a mathematical generalization of this problem in the case where two of the primaries orbit the Sun^15^. W.S.Koon et al.^16^ used two coupled CR3BP to show that the costs for transfers between the Earth and the Moon can be lowered if the Sun is taken into account.

In fact, among those described above, the CR3BP and the specific model developed by^3^—known as the bi-circular four body problem (bi-circular 4BP)—have been largely used to evaluate the costs for transfers between the Earth and the Moon. The first known work of this kind^17^ uses the perturbation of the Sun to make a ballistic capture in a transfer from the Earth to the Moon, which could not be done using the CR3BP. Costs of this transfer were evaluated using the CR3BP^18^ and the bi-circular 4BP^19^ under the same conditions. When comparing the results of these studies for short time transfers (up to 7 days), the lowest cost for the CR3BP was 3951.57 m/s, while the lowest cost for the bi-circular 4BP was 3949.53 m/s. Later, these values were lowered using different techniques to 3946.93 m/s for the CR3BP^20^ (using the Theory of Functional Connections technique) and 3944.8 m/s for the bi-circular 4BP^21^ (using a direct transcription and multiple shooting method). Many other investigations in the Earth to Moon transfers have been done using the bi-circular 4BP, for instance, the ones shown in^22–25^.

The Jacobi integral and the zero velocity surfaces are important properties of the CR3BP, because they can show the boundaries of the motion. The perturbation due to the Sun over these zero velocity surfaces is investigated in^3^. For instance, it is shown that the zero velocity surface that passes through the Lagrangean point $L_1$ does not meet the zero velocity surface that passes through $L_2$ in a Sun–Earth–Moon system. Thus, it was concluded that the boundaries of the motion in the bi-circular 4BP are close to these boundaries in the CR3BP for this system. Regions of stability for the motion was investigated in^26^.

In this paper, a different approach is done. Instead of analyzing the stability through the zero velocity surfaces, a direct comparison of the bi-circular 4BP with respect to the CR3BP is done. This comparison is possible due to the perturbative term added to CR3BP, which is obtained from both the gravitational influence of the Sun and the motion of the other bodies around it. This perturbative term, which is defined as the difference between the three and four body problems, was used to investigate the connection between the two independent pairs of CR3BP (one is the Sun–Earth system and the other is the Earth–Moon system) and the bi-circular four body problem^27^. The transfers are evaluated there from Lyapunov orbits around the Lagrangean fixed point of the Sun–Earth to the Lagrangean fixed point of the Earth–Moon systems. In comparison, in this work, a detailed analysis is done in the region between the two smaller primaries, instead. This is a region of interest for travels between and around the smaller primaries. The influence of the perturbation in this region is analyzed both analytically and numerically. The new analytical results show that the perturbation is null at the barycenter, and it has specific patterns around it. For instance, it is shown that the perturbation is linearly proportional to the displacement from the barycenter for a specific region and its magnitude grows faster with the *x* and *y* coordinates than it grows with the *z* coordinate in the region around the barycenter. Furthermore, the costs of short time transfers between the smaller primaries for the CR3BP and the bi-circular 4BP are numerically evaluated and compared for the Sun–Earth–Moon, Sun–Mars–Phobos, Sun–Saturn–Titan, and Sun–Ida–Dactyl systems. The divergence (and the convergence) of both models are revealed through some new indices related to the magnitude of the perturbation over the magnitude of the gravity of the moon. The influence of the parameters over the perturbation is also shown.

The results can be used in the choice of the system to be adopted for evaluations of the costs of transfers. It can be useful, for instance, in automatized process of evaluations of costs, since the indices shown here can reveal the regions in which the more complete model (the bi-circular 4BP) offers more fuel gain in comparison with the more simplified one (the CR3BP).

Both models are described in “Mathematical models” section. The numerical and analytical results are shown in “Results” section, before the conclusions, which are available in the last section.

## Mathematical models

In this section, the mathematical tools and the two models adopted in this research are shown and compared.

### The bi-circular bi-planar four body problem

A vector ${{\varvec{h}}^*}$ connects the center of an inertial frame of reference to the center of a rotating frame of reference. The Sun is located at the center of the inertial frame, and the barycenter of a $M_e$-moon system is located at the center of the rotating frame of reference. The inertial frame of reference is denoted by a star (*), for clarity purpose. Thus, the position of a particle in the rotating frame of reference (${{\varvec{r}}}$) with respect to the inertial one (${{\varvec{r}}^*}$) is

1 $$\begin{aligned} {{\varvec{r}}}={{\varvec{r}}}^*-{{\varvec{h}}^*} \end{aligned}$$

The acceleration can be written as^28^

2 $$\begin{aligned} \frac{\; \text {d}^2 {{\varvec{r}}}}{\; \text {d}t^2}=\frac{\; \text {d}^{*2} {{\varvec{r}}^*}}{\; \text {d}t^2}- {{\varvec{\omega }}} \times \left( {{\varvec{\omega }}} \times {{\varvec{r}}}\right) - 2 \, {{\varvec{\omega }}} \times \frac{\; \text {d}{{\varvec{r}}}}{\; \text {d}t} - \frac{\; \text {d}^{*} {{\varvec{\omega }}}}{\; \text {d}t} \times {{\varvec{r}}} - \frac{\; \text {d}^{*2} {{\varvec{h}}^*}}{\; \text {d}t^2} \end{aligned}$$

where ${{\varvec{\omega }}}$ is the angular velocity of the rotating frame. The derivatives with no star ($\frac{\; \text {d}}{\; \text {d}t}$) are taken in the rotating frame, i.e. it does not take into consideration the motion of its base, while the derivatives with star ($\frac{\; \text {d}^* }{\; \text {d}t}$) are taken in the inertial frame. Hence, the equation of motion of a particle written in the rotating frame of reference is

3 $$\begin{aligned} \frac{\; \text {d}^2 {{\varvec{r}}}}{\; \text {d}t^2} + 2 \, {{\varvec{\omega }}} \times \frac{\; \text {d}{{\varvec{r}}}}{\; \text {d}t} + {{\varvec{\omega }}} \times \left( {{\varvec{\omega }}} \times {{\varvec{r}}}\right) +\frac{\; \text {d}^{*} {{\varvec{\omega }}}}{\; \text {d}t} \times {{\varvec{r}}} + \frac{\; \text {d}^{*2} {{\varvec{h}}^*}}{\; \text {d}t^2} = {{\varvec{a}}} \end{aligned}$$

where ${{\varvec{a}}}$ is the acceleration acting over the particle.

**Figure 1:**
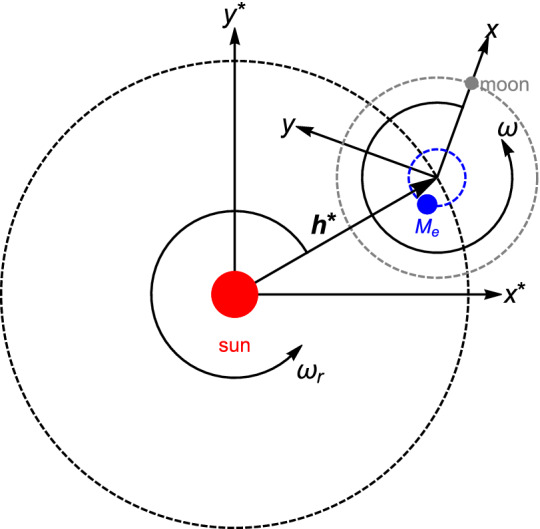
The two frames of reference in the Sun-$M_e$-moon system.

In the bi-planar, bi-circular four-body problem, the body $M_e$ and the moon rotate in a circular orbit around their barycenter, which in turn rotates in a circular orbit around the Sun. Moreover, the two orbital planes coincide with each other. The two frames of reference can be seen in Fig. 1.

The barycenter of the $M_e$-moon rotates in a circular motion around the Sun, according to

4 $$\begin{aligned} {{\varvec{h}}^*}=\Big (R_s \cos {\theta _1},R_s \sin {\theta _1},0\Big ) \end{aligned}$$

where $R_s$ is the constant distance between the Sun and the barycenter, and $\theta _1=(\omega _r t+\gamma -\pi )$ is the angle between ${{\varvec{h}}^*}$ and the positive side of the $x^*$ axis, where $\gamma -\pi$ is an initial phase and $\omega _r$ is the angular velocity of ${{\varvec{h}}^*}$.

The second derivative of ${{\varvec{h}}^*}$ with respect to the inertial frame, written in the rotating frame of reference is

5 $$\begin{aligned} \dfrac{\; \text {d}^{*2} {{\varvec{h}}^*}}{\; \text {d}t^2} = \dfrac{\mu _s}{R_s^2} \Big ( \cos {(\omega _s t + \gamma )} , \sin {(\omega _s t + \gamma )},0\Big ), \end{aligned}$$

where $\mu _s$ is the gravitational parameter of the Sun and $\omega _s=\omega _r-\omega$.

The vectors that locate the spacecraft with respect to $M_e$, the moon, and the Sun are ${{\varvec{r}}}_e$, ${{\varvec{r}}}_m$, and ${{\varvec{r}}}_s$, respectively. The first two are given by ${{\varvec{r}}}_e={{\varvec{r}}} + d_e \hat{{\varvec{x}}}$ and ${{\varvec{r}}}_m={{\varvec{r}}} - d_m \hat{{\varvec{x}}}$, where $\hat{{\varvec{x}}}$ is an unitary vector along the *x* axis, $d_e = R \mu _m/(\mu _e + \mu _m)$ and $d_m = R \mu _e /(\mu _e + \mu _m)$ are the distances between the barycenter and $M_e$ and the barycenter and the moon, respectively, where $\mu _e$ and $\mu _m$ are the gravitational parameters of $M_e$ and the moon, respectively. The last one is given by

6 $$\begin{aligned} {{\varvec{r}}}_s={{\varvec{r}}}-{{\varvec{R}}}_s \end{aligned}$$

where ${{\varvec{R}}}_s=R_s\Big ( \cos {\theta _2},\sin {\theta _2},0\Big )$ locates the Sun in the rotating frame, where $\theta _2=\theta _2(t)=\omega _s t+\gamma$.

Hence, the equation of motion (3), according to the bi-circular bi-planar four body problem, becomes

7 $$\begin{aligned} \frac{\; \text {d}^2 {{\varvec{r}}}}{\; \text {d}t^2} + 2 \, {{\varvec{\omega }}} \times \frac{\; \text {d}{{\varvec{r}}}}{\; \text {d}t} + {{\varvec{\omega }}} \times \left( {{\varvec{\omega }}} \times {{\varvec{r}}}\right)&= - \dfrac{\mu _e}{r_e^3} \, {{\varvec{r}}}_e - \dfrac{\mu _m}{r_m^3} \, {{\varvec{r}}}_m\nonumber \\&\quad -\frac{\mu _s}{r_s^3}{{\varvec{r}}}_s-\frac{\mu _s}{R_s^2}\Big (\cos {\theta _2}, \sin {\theta _2},0\Big ). \end{aligned}$$

### The circular restricted three body problem

In the case where the Sun is not taken into consideration and the center of the inertial frame of reference is located at the barycenter of the $M_e$-moon system, the equation of motion becomes

8 $$\begin{aligned} \frac{\; \text {d}^2 {{\varvec{r}}}}{\; \text {d}t^2} + 2 \, {{\varvec{\omega }}} \times \frac{\; \text {d}{{\varvec{r}}}}{\; \text {d}t} + {{\varvec{\omega }}} \times \left( {{\varvec{\omega }}} \times {{\varvec{r}}}\right) = - \dfrac{\mu _e}{r_e^3} \, {{\varvec{r}}}_e - \dfrac{\mu _m}{r_m^3} \, {{\varvec{r}}}_m \end{aligned}$$

The perturbation due to the gravity of the moon is

9 $$\begin{aligned} {{\varvec{p}}}_m=- (\mu _m/r_m^3) \, {{\varvec{r}}}_m \end{aligned}$$

It should be noted that the motion is Keplerian when this term is null in Eq. (8).

### Perturbation due to the gravity of the Sun

Note that, in the case where $\mu _s=0$, Eq. (7) is reduced to the planar circular restricted three-body problem for the $M_e$-moon-satellite system given by Eq. (8), which does not take into consideration the influence of the Sun. Thus, the perturbation of the Sun over the satellite is given by the last two terms of Eq. (7), which can be written as

10 $$\begin{aligned} {{\varvec{p}}}_s=-\frac{\mu _s}{r_s^3}{{\varvec{r}}}_s-\frac{\mu _s}{R_s^3}{{\varvec{R}}}_s \end{aligned}$$

Note that this perturbation does not represent only the acceleration due to the gravity of the Sun, it is a pseudo specific force instead. The second term on the right side comes from the fact that the rotating frame of reference (and also the pair $M_e$-moon) is in an accelerated motion around the Sun. On the other side, it can be seen as a perturbation in the CR3BP due to the presence of the Sun.

### Orbit transfer using a bi-impulsive maneuver

Comparisons between both models will be shown for several bodies in the Solar system, with different masses and distances. This is done through an important subject of the astrodynamics, which is the costs associated to a transfer of a spacecraft from an orbit around a massive body to another orbit close to its moon.

The transfer is done in the following way: a spacecraft is initially in a circular orbit of radius $r_0$ around the massive body $M_e$, when a first impulse is applied to it at a point *A* of this orbit. This impulse is applied such that the spacecraft travels to a second point *B* close to the moon in a given time of flight. The distance between *B* and the moon is $\rho _0$. A second impulse is applied at the point *B* such that the spacecraft enters in a circular orbit around the moon. The magnitude of the differences of the velocity of the spacecraft before and after the first impulse at point *A* plus the magnitude of the difference of the velocity of the spacecraft before and after the second impulse at point *B* is the cost of the transfer.

This problem is known as the “two point boundary value problem”, i.e. the maneuver is constrained such that the position of the spacecraft at the initial time is *A* and its position at the final time is *B*. The difference between the final and initial times is the time of flight, which is assumed to be a given parameter of the transfer. The solution of this problem is obtained using the Theory of Functional Connections^29,30^. This is a very efficient approach to solve both linear and and nonlinear differential equations^31,32^ subjected to two constraints in the positions—the “two point boundary value problem”. More details in the use of the Theory of Functional Connections to solve the orbit transfer problem can be seen in^20^. The numerical evaluations was done using the Python language combined with a TFC Python module freely available in^33^. An algorithm is used to find local minima around the neighborhood of a point in the space of parameters, which are varied each at a time. The costs in this neighborhood are compared and the parameters associated to the lowest value of the cost are selected as the optimal local maneuver. This is done for all of the parameters, several times. The step is reduced for each successive iteration, until the variation of the cost after each step is no more than $10^{-3}~\text {m/s}$, which is assumed to be our acceptable error. In this way, the minimum cost associated to a given time of flight is found through the algorithm described above.

The cost associated to the time of flight is the minimum for the given transfer time, and it is found for each of the two models: the CR3BP and the bi-circular bi-planar 4BP. In each case, the data are fitted using Legendre’s polynomials with its 20 first terms. The resulting curves are the costs as function of the time of flight for the 3BP (named $\textit{cost}_{3BP}$) and the 4BP (named $\textit{cost}_{4BP}$). The *cost gain* is defined as the cost required by the 3BP minus the cost required by the 4BP, given by

11 $$\begin{aligned} \textit{cost}\,\textit{gain} = \textit{cost}_{3BP}-cost_{4BP}. \end{aligned}$$

Thus, the *cost gain* shows the gain of the 4BP with respect to the 3BP in terms of variation of velocity required by the maneuver. The relative cost gain is defined as the cost gain divided by the cost associated to the 3BP, according to

12 $$\begin{aligned} \textit{relative}\,\textit{cost}\,\textit{gain} = (\textit{cost}_{3BP}-\textit{cost}_{4BP})/\textit{cost}_{3BP}. \end{aligned}$$

Thus, this ratio compares the cost gain with the cost of the transfer. Finally, the relative cost gain per time of flight is defined as the relative cost gain divided by the respective time of flight:

13 $$\begin{aligned} \textit{relative}\,\textit{cost}\,\textit{gain}\,\textit{per}\,\textit{time}\,\textit{of}\,\textit{flight} = \textit{relative}\,\textit{cost}\,\textit{gain}/\textit{time of flight}. \end{aligned}$$

This is an important factor, because longer times of flight tend to accumulate the effect of the perturbation over the costs, and this index tends to cancel this dependency.

## Results

The results obtained from simulations using the two models described above are shown next.

### Perturbation at the barycenter

In the case where the spacecraft is located at the barycenter, its position is given by ${{\varvec{r}}}={{\varvec{0}}}$, where ${{\varvec{0}}}$ is the null vector. Hence, according to Eq. (6), its position with respect to the Sun is ${{\varvec{r}}}_s=-{{\varvec{R}}}_s$. Using this result, the perturbation given by Eq. (10) becomes ${{\varvec{p}}}_s={{\varvec{0}}}$. Hence, there is no perturbation from the Sun at the barycenter of the Earth and the Moon—both models are identical at this point.

### Perturbation on a sphere around the Sun

The perturbation given by Eq. (10) can be written as

14 $$\begin{aligned} {{\varvec{p}}}_s=-\frac{\mu _s}{r_s^3}{{\varvec{r}}}+\frac{\mu _s}{r_s^3}{{\varvec{R}}}_s-\frac{\mu _s}{R_s^3}{{\varvec{R}}}_s \end{aligned}$$

In the case where the spacecraft is located in a sphere around the Sun of radius $R_s$, the magnitude of ${{\varvec{r}}}_s$ is given by $r_s=R_s$. Hence, Eq. (14) becomes

15 $$\begin{aligned} {{\varvec{p}}}_s=-\frac{\mu _s}{R_s^3}{{\varvec{r}}} \end{aligned}$$

In this case, the perturbation due to the Sun is directed toward the barycenter and its magnitude is proportional to the displacement.

### Perturbation around the barycenter

The magnitude of the first term on the right side of Eq. (14) is much smaller than each of the last two terms. On the other side, the last two terms tend to cancel each other for $r_s$ close to $R_s$, which is the case considered now. Hence, neither term can be neglected. A series expansion of Eq. (10) around the position of the barycenter $\big (0,0,0\big )$—neglecting terms of order greater than one and crossed terms on *x*, *y*, *z*—is given by

16 $$\begin{aligned} {{\varvec{p}}}_s\approx \frac{\mu _s}{R_s^3}\Bigg (\frac{(3 x \cos (2 \theta _2)+3 y \sin (2 \theta _2)+x)}{2},\frac{(3 x \sin (2 \theta _2)-3 y \cos (2 \theta _2)+y)}{2},-z\Bigg ) \end{aligned}$$

Hence, its magnitude is

17 $$\begin{aligned} \Vert {{\varvec{p}}}_s\Vert \approx \frac{\mu _s}{R_s^3}\frac{1}{\sqrt{2}}\sqrt{ \left( 3 \cos (2 \theta _2) \left( x^2-y^2\right) +6 x y \sin (2 \theta _2)+5 x^2+5 y^2+2 z^2\right) } \end{aligned}$$

For a constant *z*, the dominant term inside the square root is $(5x^2+5y^2)$. Thus, constant values of the magnitude of the perturbation are distorted concentric circles around the barycenter. This distortion depends on the value of $\theta _2$, or the time, because $\theta _2$ is a function of time. In the space, the term $2z^2$ is taken into account, and constant values of this magnitude are distorted spherical shells around the barycenter, and it tends to increase faster with respect to *x* and *y* in comparison to its dependence with *z*.

### Relative perturbation

The relative perturbation is defined as the ratio between the magnitude of the perturbation and the magnitude of the gravitational attraction of the moon, thus it is given by $\Vert {{\varvec{p}}}_s\Vert /\Vert {{\varvec{p}}}_m\Vert$. This ratio shows a very important relationship between these two variables, because ${{\varvec{p}}}_m$ is the perturbation due to the moon over a Keplerian orbit, and ${{\varvec{p}}}_s$ is the perturbation over the orbit obtained using the CR3BP. The larger this number, the more important is the perturbation due to the Sun in comparison with the perturbation due to the moon over a Keplerian orbit.

The relative perturbation is negligible around the barycenter (because $\Vert {{\varvec{p}}}_s\Vert =0$ at the barycenter) and close to the moon (because $\Vert {{\varvec{p}}}_m\Vert$ is much larger when the position tends to ${{\varvec{r}}}\approx d_m \hat{x}$). On the other hand, this quantity depends on several parameters between the barycenter and the moon.

### The Sun–Earth–Moon system

The results are shown below in this section for the values of the parameters of the Sun–Earth–Moon system shown in Table 1.

**Table 1:** Values of the parameters for the Sun–Earth–Moon^34^.

$$\mu _s=1.3237395128595653\times 10^{20}\,\text {m}^3/\text {s}^2$$	
$$\mu _e=3.975837768911438\times 10^{14}\,\text {m}^3/\text {s}^2$$	
$$\mu _m=4.890329364450684\times 10^{12}\,\text {m}^3/\text {s}^2$$	
$$R=3.84405000\times 10^{8}\,\text {m}$$	
$$R_s=1.49460947424915\times 10^{11}\,\text {m}$$	
$$\omega =2.66186135\times 10^{-6}\,1/\text {s}$$	

**Figure 2:**
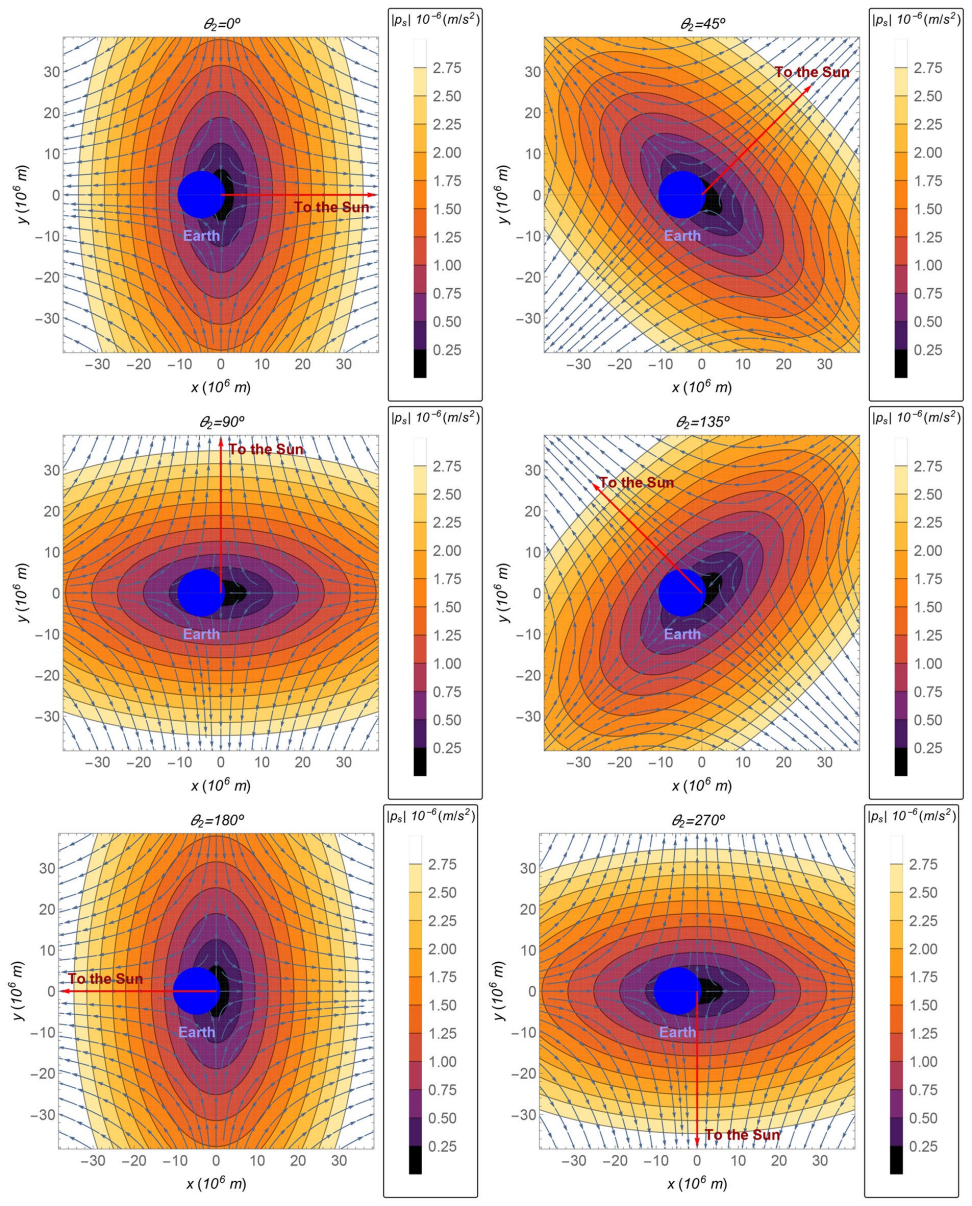
The magnitude of ${{\varvec{p}}}_s$ as a function of the coordinates, in the plane $z=0$, for the following values of the angle $\theta _2$: $0^{\circ }$. 45$^{\circ }$, 90$^{\circ }$, 135$^{\circ }$, 180$^{\circ }$, and 270$^{\circ }$ (from left to right and, then, up to down).

**Figure 3:**
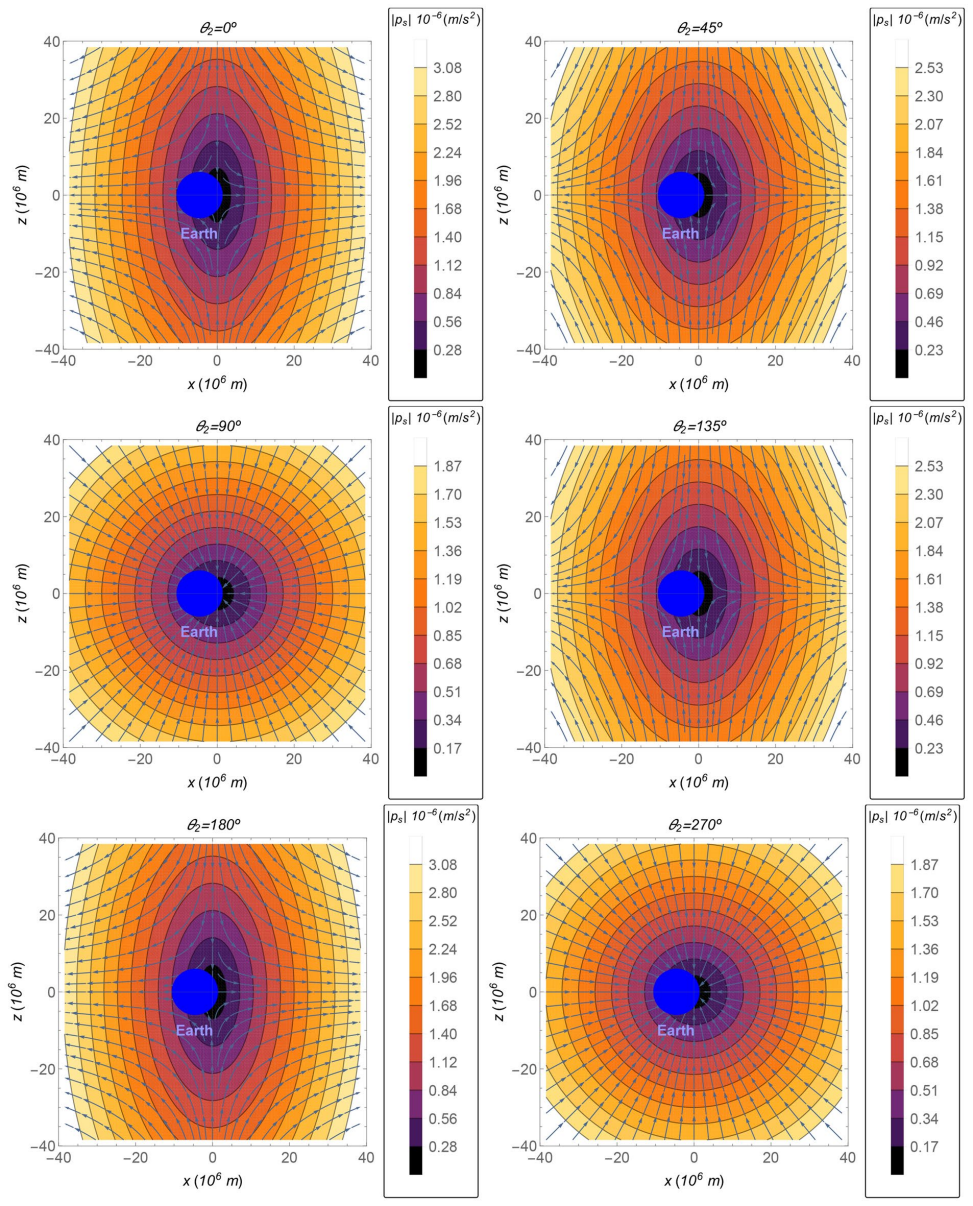
The magnitude of ${{\varvec{p}}}_s$ as a function of the *x*–*z* coordinates, in the plane $y=0$, for the following values of the angle $\theta _2$: 0$^{\circ }$. 45$^{\circ }$, 90$^{\circ }$, 135$^{\circ }$, 180$^{\circ }$, and 270$^{\circ }$ (from left to right and, then, up to down).

The direction of the perturbation ${{\varvec{p}}}_s$ is represented by the streamlines shown in Fig. 2, as function of the coordinates, for $\theta _2=0^{\circ }$ (up left), $\theta _2=45^{\circ }$ (up right), $\theta _2=90^{\circ }$ (middle left), $\theta _2=135^{\circ }$ (middle right), $\theta _2=180^{\circ }$ (down left), and $\theta _2=270^{\circ }$ (down right). The magnitude of the perturbation ${{\varvec{p}}}_s$ is shown by the gradient color, from dark purple for lower values of the magnitude of the perturbation to brighter yellow for larger values. Although the angle $\theta _2$ is a function of time ($\theta _2=\theta _2(t)=\omega _s t+\gamma$), the effects on the perturbation of its oscillation from 0 to 2$\pi$ can be seen by the drawn of the discrete sequence of its values shown in Fig. 2. An analogous drawn is done in Fig. 3 for the perturbation, as a function of *x* and *z*, for $y=0$. The direction of the Sun is perpendicular to the *x*–*z* plane in the case where the relative position of the Sun is $\theta _2=90^{\circ }$ or $\theta _2=270^{\circ }$. In these cases, the direction of the perturbation is toward the barycenter. Note that, in the regions where the position is such that $r_s\approx R_s$, the perturbation is approximated by the same value as the one it has on a sphere around the Sun, shown in the subsection above, i.e. ${{\varvec{p}}}_s\approx -\mu _s{{\varvec{r}}}/R_s^3$. This behavior can be seen in Fig. 2 for the regions close to a straight line that crosses the barycenter and is perpendicular to the direction of the Sun. Furthermore, this behavior can also be seen in Fig. 3, in the case where $\theta _2=90^{\circ }$ or $\theta _2=270^{\circ }$. Note that the sphere given by the equation $r_s=R_s$ yields to $y=0$ in regions close to the Earth. Hence, the perturbation is proportional to the negative of the position for these cases ($\theta _2=90^{\circ }$ or $\theta _2=270^{\circ }$), according to Eq. (15), with a good accuracy.

**Figure 4:**
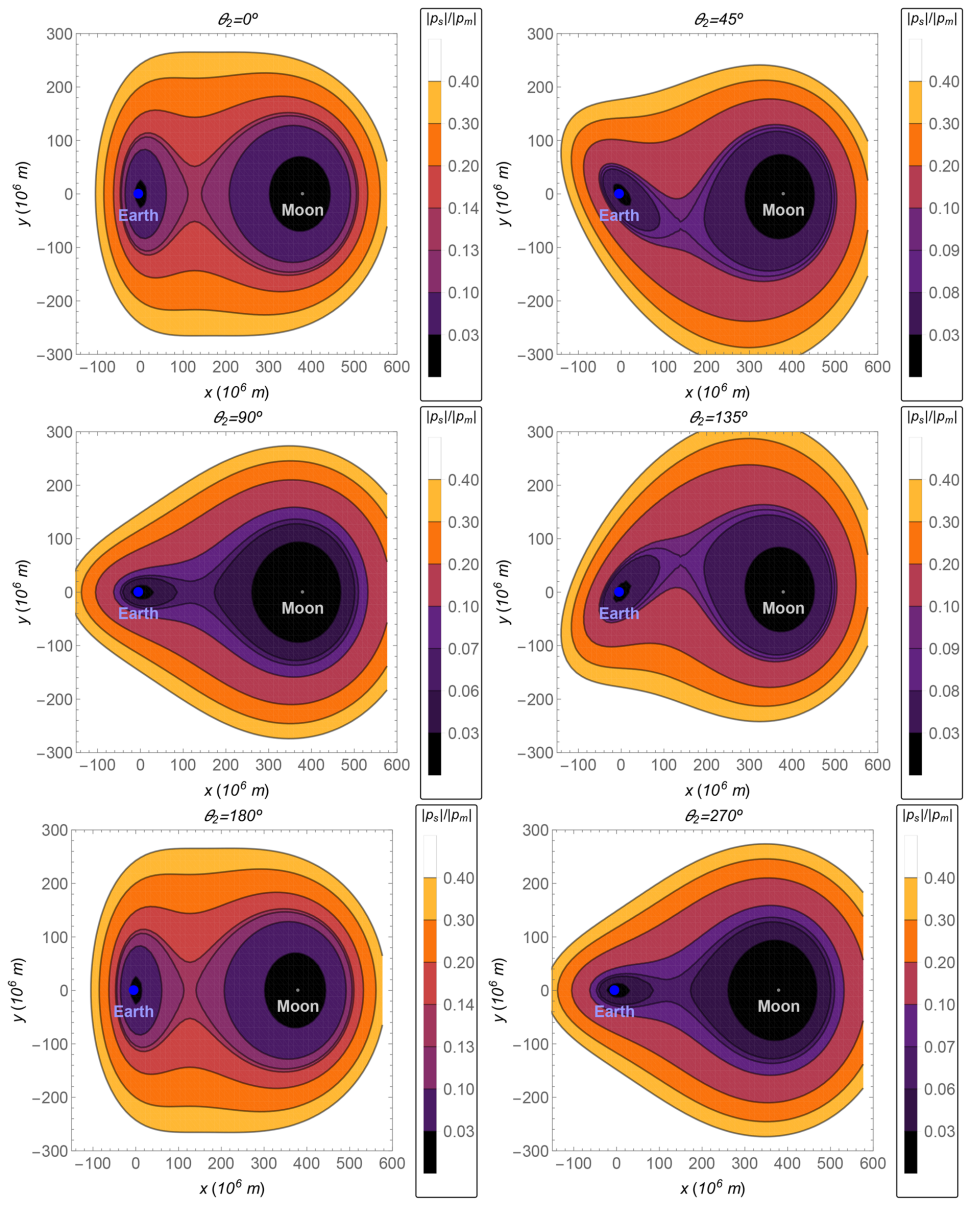
The ratio $\Vert {{\varvec{p}}}_s\Vert /\Vert {{\varvec{p}}}_m\Vert$ as a function of the *x*–*y* coordinates, in the plane $z=0$, for several values of the angle $\theta _2$.

The relative perturbation defined in “Relative perturbation” section is shown in Fig. 4. In the case, where the position of the Sun is $\theta _2=0^{\circ }$ or $\theta _2=180^{\circ }$, the perturbation given by ${{\varvec{p}}}_s$ is stronger compared with other values for $\theta _2$. The magnitude of the perturbation is at least 1/10th of the acceleration due to the gravitational attraction of the Moon over the spacecraft for any trajectory from the Earth to the Moon. It is at least 1/20th for other values of $\theta _2$.

**Figure 5:**
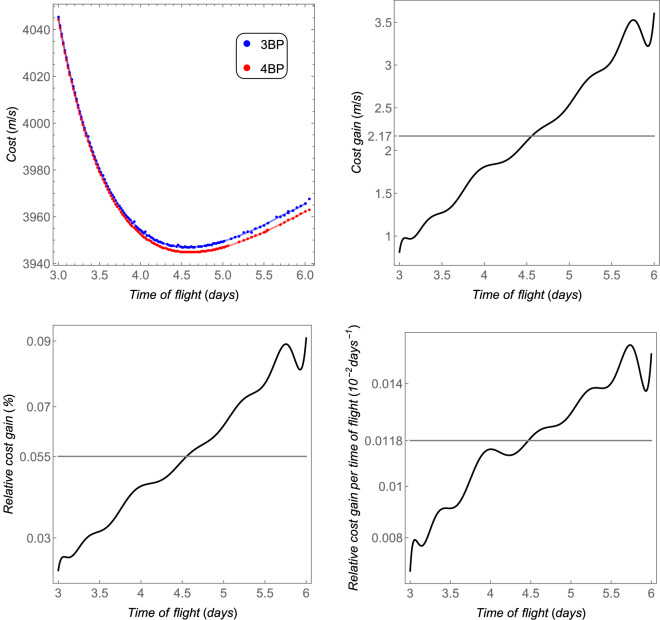
The total costs and the equivalent fuel savings of the 4BP in comparison with the 3BP for the Earth–Moon system. The horizontal gray thicker straight lines are the average values.

The *cost* as a function of the time of flight (defined in “Orbit transfer using a bi-impulsive maneuver” section) is shown in the upper left side of Fig. 5, for an initial orbit of 167 km of altitude around the Earth to another circular orbit of 100 km of altitude around the Moon. Note that this is the minimum cost for the respective time of flight. The overall minima of these costs are 3946.92 m/s, which occurs for a time of flight of 4.58 days in the case of the 3BP, and 3944.83 m/s, for a time of flight of 4.6 days in the case of the 4BP. These results are in agreement with the ones found in^20^. The red and blue curves are fit to these data using Legendre’s polynomials with 20 terms. The *cost gain* is shown in the upper right side of Fig. 5. Both models tend to show the same costs for shorter time transfers, but they diverge for longer times, due to the cumulative effect of the perturbation ${{\varvec{p}}}_s$. Note that this perturbation may increase or decrease the *cost*, depending on the position of the Sun. Only the best result (the lower one) is shown, because these costs are the minima found through the algorithm explained in “Orbit transfer using a bi-impulsive maneuver” section. The *cost gain* may vary from 1 m/s to 3.5 m/s, and the average is shown in the gray straight line. The *relative cost gain* goes from 0.03 % to 0.1%. Finally, the *relative cost gain per time of flight* is of the order of $10^{-4}$/days.

### The Sun–Mars–Phobos system

**Figure 6:**
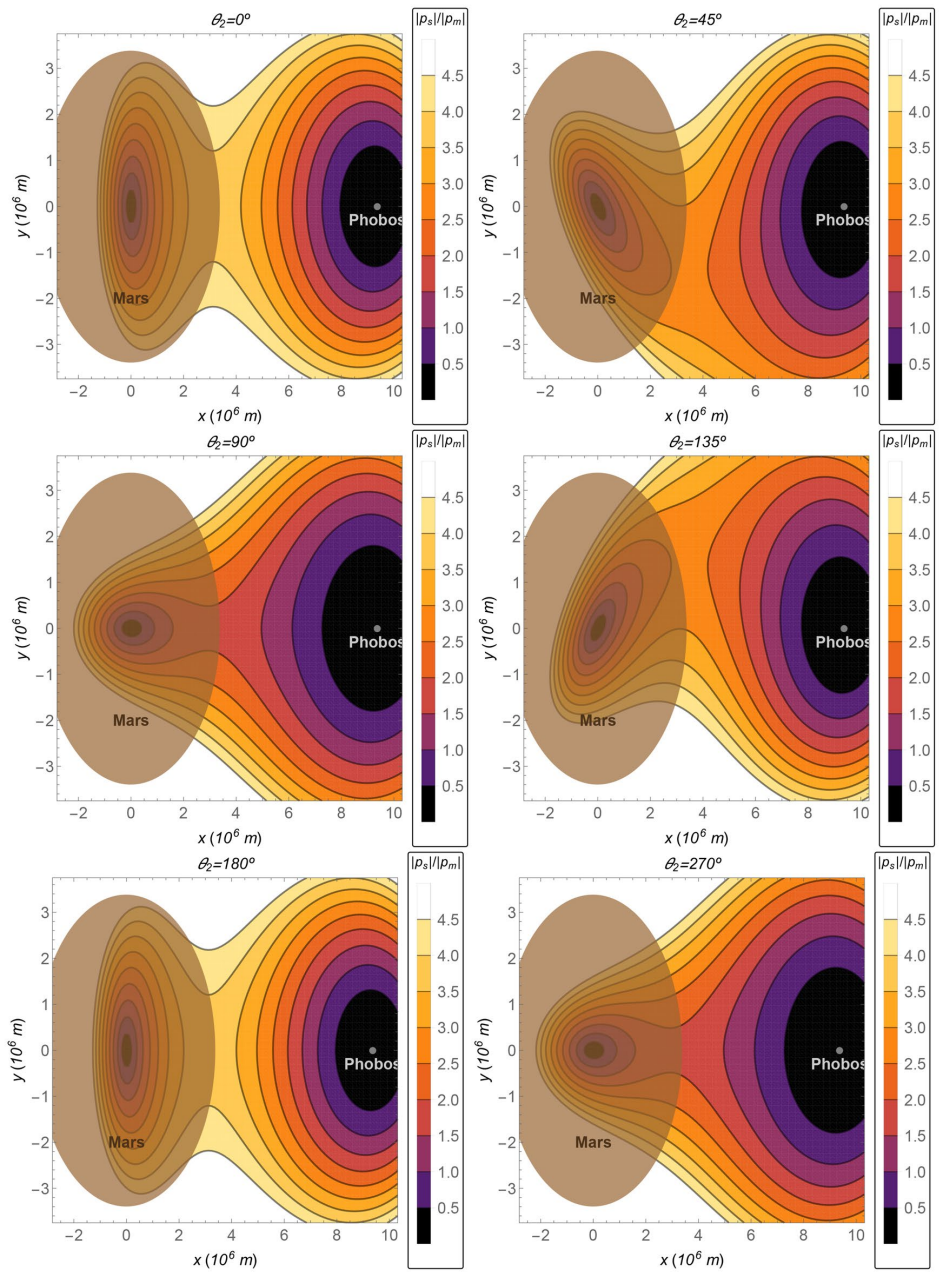
The Mars–Phobos system. The ratio $\Vert {{\varvec{p}}}_s\Vert /\Vert {{\varvec{p}}}_m\Vert$ as a function of the *x*–*y* coordinates, in the plane $z=0$, for several values of the angle $\theta _2$.

In this section, the Sun–Mars–Phobos and the Mars–Phobos systems are analyzed and compared. The parameters for these systems are shown in Table 2. The relative perturbation in the plane $x-y$ can be seen in Fig. 6. Note that, in this case, the barycenter of the pair Mars–Phobos is almost in the center of Mars, far from its surface. On the other hand, the magnitude of the perturbation due to the gravity of Phobos is very small. Hence, although the distance Mars–Phobos is small and the region around the pair is close to the barycenter (where $\Vert {{\varvec{p}}}_s\Vert$ is null), the relative perturbation is one order of magnitude higher in comparison with the Earth–Moon case. Thus, the motion described by the CR3BP converges with the one described by the bi-planar bi-circular 4BP only in a small region around Phobos. This comparison also helps to understand the importance of each body in the dynamics.

**Table 2:** Values of the parameters for the Sun–Mars–Phobos system^34–36^.

$$\mu _s=1.3237395128595653\times 10^{20}\,\text {m}^3/\text {s}^2$$	
$$\mu _e=4.28309084016 \times 10^{13}\,\text {m}^3/\text {s}^2$$	
$$\mu _m=7.20811872 \times 10^{5}\,\text {m}^3/\text {s}^2$$	
$$R=9.376\times 10^{6}\,\text {m}$$	
$$R_s=1.523679\,\text {AU}$$	
$$\omega =\sqrt{\mu _e/R^3}$$	

**Figure 7:**
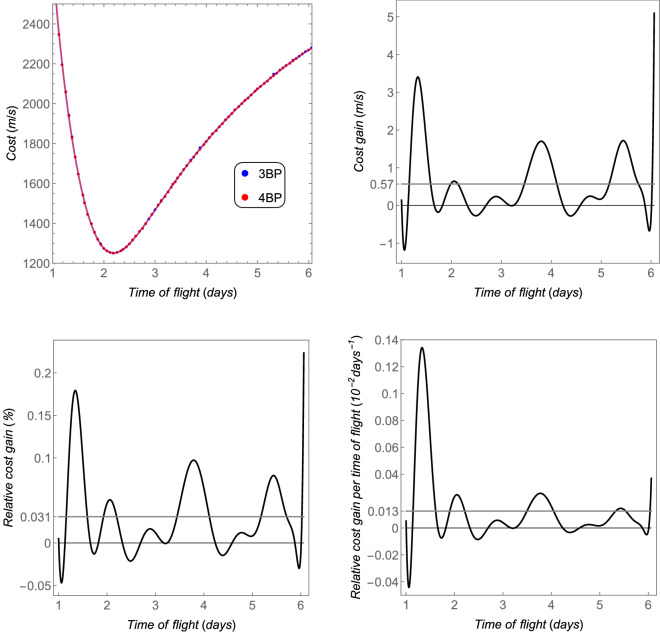
The total costs and the equivalent fuel savings of the 4BP in comparison with the 3BP for the Sun–Mars–Phobos system. The horizontal ticker gray line is the average value.

The minimum cost as a function of the time of flight is shown in Fig. 7 (up left part) for a transfer from an initial circular orbit around Mars of 167 km of altitude (above the Mars radius of 3389.5 km) to a final circular orbit around Phobos with 10 km in altitude (21.2667 km from its center). The *cost gain* is shown in the up right side, the *relative cost gain* is shown in the down left side, and the *relative cost gain per time of flight* is shown in the down right side of the same figure. Although the *cost gain* is of the same order of magnitude in comparison to the Earth–Moon system, the *relative cost gain per time of flight* is one order higher. This result is in agreement with the comparison of the relative perturbation, which is also one order of magnitude greater than the one in the Earth–Moon system (see Figs. 6 and 4).

### The Sun–Saturn–Titan system

**Figure 8:**
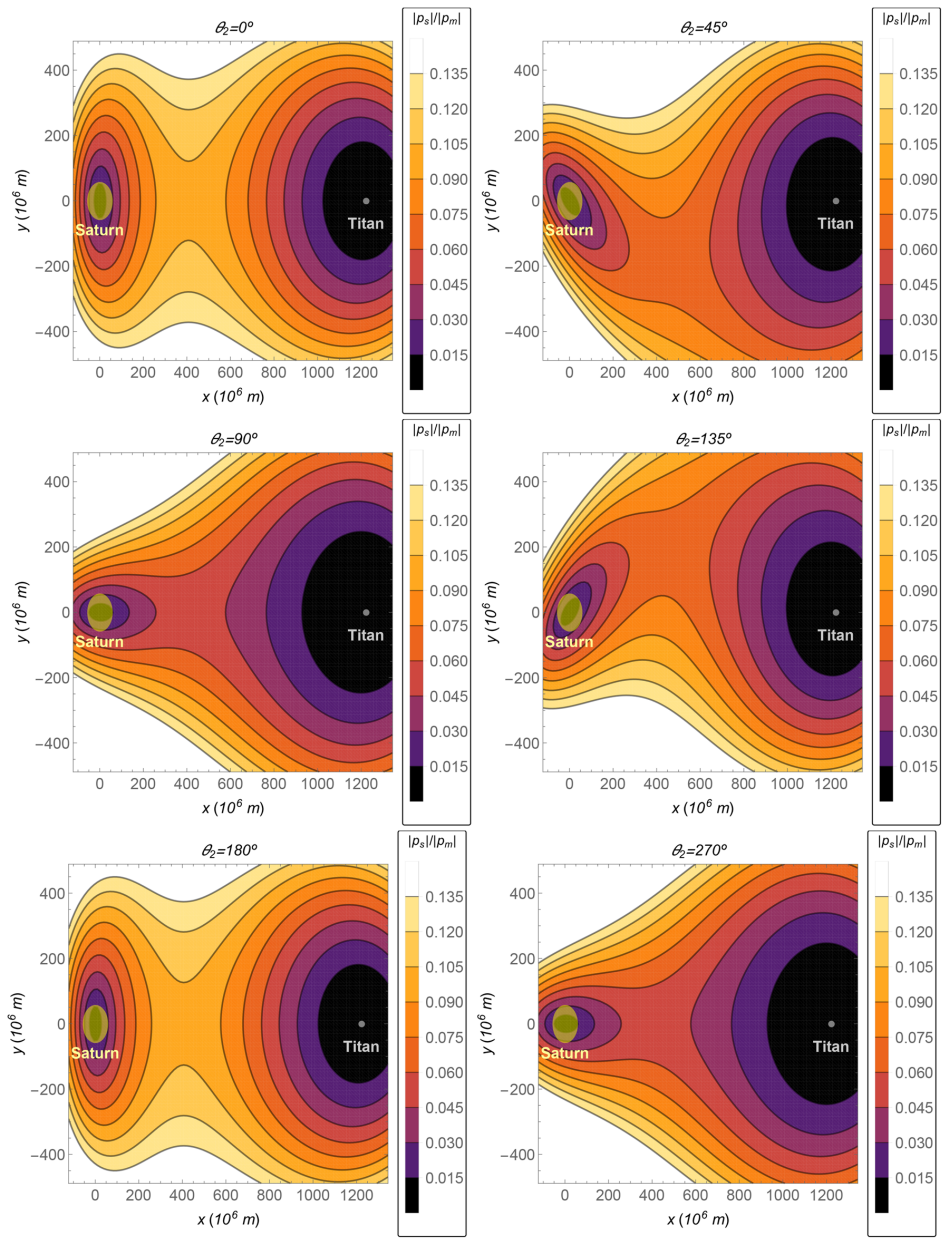
The Saturn–Titan system. The ratio $\Vert {{\varvec{p}}}_s\Vert /\Vert {{\varvec{p}}}_m\Vert$ as a function of the *x*–*y* coordinates, in the plane $z=0$, for several values of the angle $\theta _2$.

In this subsection, the relative perturbation is shown in Fig. 8 for the Sun–Saturn–Titan system, whose parameters can be seen in Table 3. The relative perturbation is also presented for several values of the relative position of the Sun given by $\theta _2$. Note that it is about one order of magnitude lower than the value for the case of the Earth–Moon system and almost two orders of magnitude lower than the Mars–Phobos case.

**Table 3:** Values of the parameters for the Sun–Saturn–Titan system^35^.

$$\mu _s=1.3237395128595653\times 10^{20}\,\text {m}^3/\text {s}^2$$	
$$\mu _e=3.793947517 \times 10^{16}\,\text {m}^3/\text {s}^2$$	
$$\mu _m=8.977972416 \times 10^{12}\,\text {m}^3/\text {s}^2$$	
$$R=1.221870000 \times 10^9\,\text {m}$$	
$$R_s=9.5820172\,\text {AU}$$	
$$\omega =\sqrt{\mu _e/R^3}$$	

**Figure 9:**
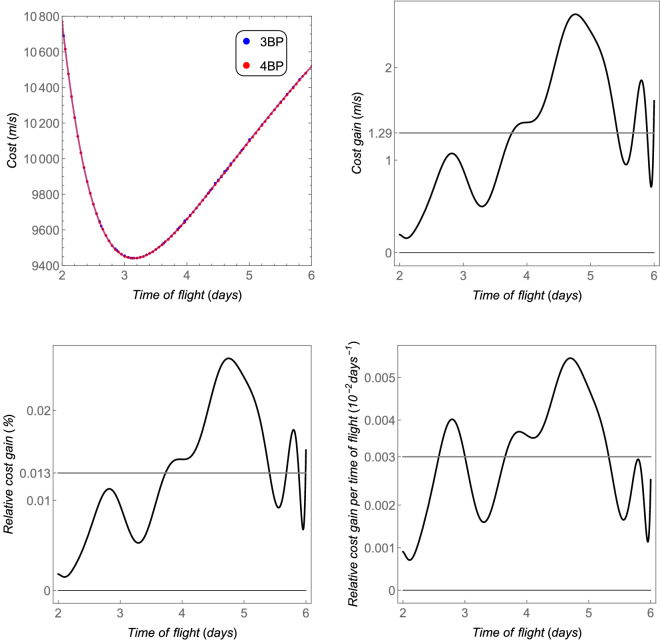
The total costs and the equivalent fuel savings of the 4BP in comparison with the 3BP for the Sun–Saturn–Titan system. The thicker gray horizontal line is the average value.

The *cost* as a function of the time of flight is shown in Fig. 9—up left side—from a circular orbit with semi-major axis of $100\times 10^6~\text {m}$ to another circular orbit around Titan with 100 km of altitude (semi-major axis equals to $2.657473\times 10^6~\text {m}$). The *cost gain*, the *relative cost gain*, and the *relative cost gain per time of flight* are also shown in this figure. Note that the distance Saturn–Titan is much larger than the distance Earth–Moon. Thus, the region of interest (which is the region where the transfer is realized) between these two main bodies is further from the barycenter, where the magnitude of the perturbation ($\Vert {{\varvec{p}}}_s\Vert$) is null. On the other side, Titan has more mass than our Moon, which makes its cost contribution to the transfer more significant. Thus, the relative perturbation for this case is lower than in the Earth–Moon system, in the respective regions of interest. The lower order of magnitude of the average relative index *cost gain per time of flight* can explain the lower relative perturbation of the Saturn–Titan in comparison with the Earth–Moon system (compare Fig. 4 with 8 and Fig. 5 with 9).

### The Sun–Ida–Dactyl system

**Figure 10:**
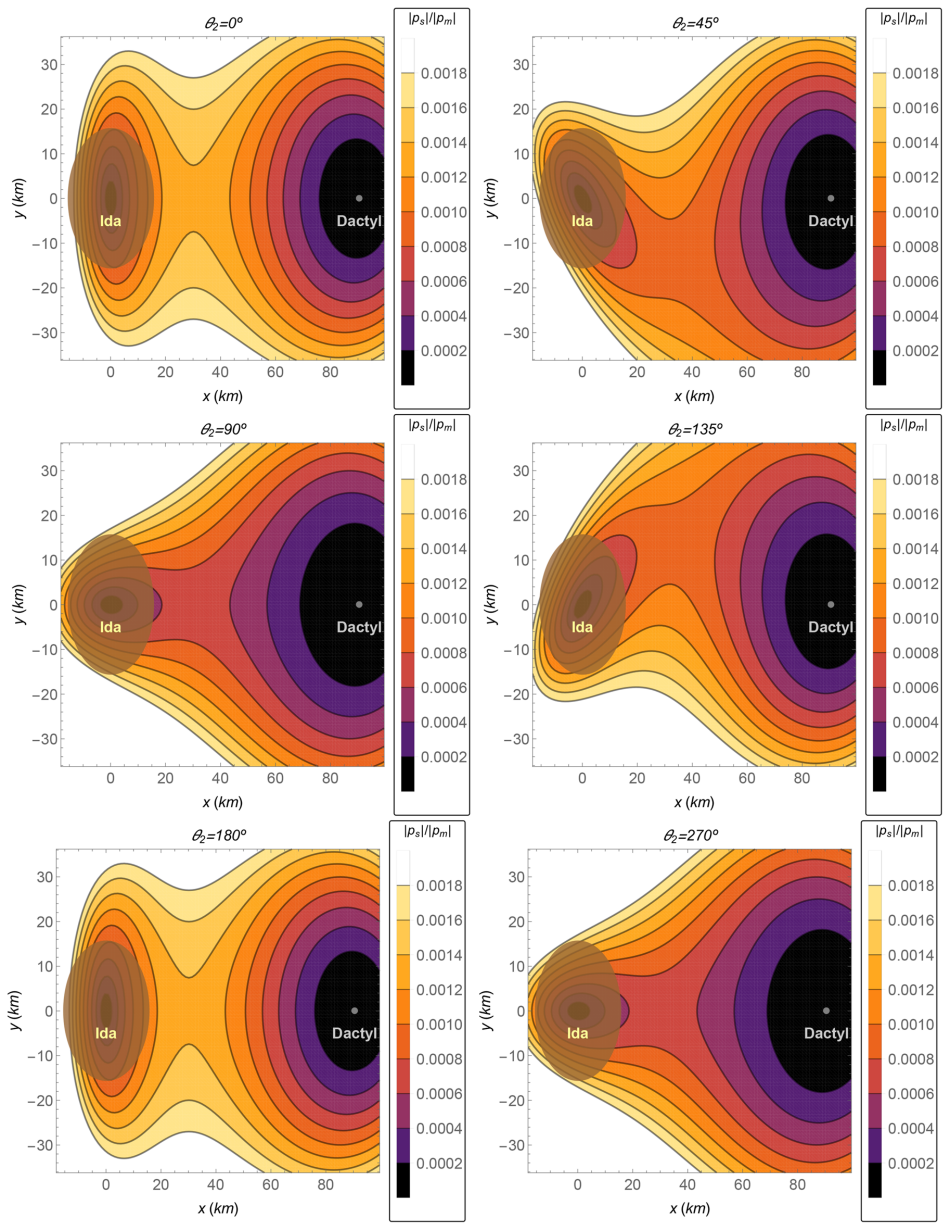
The Ida–Dactyl system. The ratio $\Vert {{\varvec{p}}}_s\Vert /\Vert {{\varvec{p}}}_m\Vert$ as a function of the *x*–*y* coordinates, in the plane $z=0$, for several values of the angle $\theta _2$.

The relative perturbation is shown in Fig. 10 for the Sun–Ida–Dactyl system, whose values of the parameters are shown in Table 4. The relative perturbation is two orders of magnitude lower than the similar values in the Earth–Moon system in the region of interest around the bodies, where the path of a short-time transfer is located. Note that the distance Ida–Dactyl is only 90.5 km, according to Table 2. The magnitude of the perturbation ($\Vert {{\varvec{p}}}_s\Vert$) is very low for this system, due to the proximity of this region to its barycenter (close to the center of Ida), which can explain the very low relative perturbation in the region around the bodies, about two orders of magnitude lower than those for the Earth–Moon system evaluated in the respective regions.

**Table 4:** Values of the parameters for the Sun–Ida–Dactyl system^34,36–38^.

$$\mu _s=1.3237395128595653\times 10^{20}\,\text {m}^3/\text {s}^2$$	
$$\mu _e=3\times 10^{6}\,\text {m}^3/\text {s}^2$$	
$$\mu _m=9\times 10^{-5}\,\mu _e$$	
$$R=90.5\,\text {km}$$	
$$R_s=2.863914916076813\,\text {AU}$$	
$$\omega =\sqrt{\mu _e/R^3}$$	

**Figure 11:**
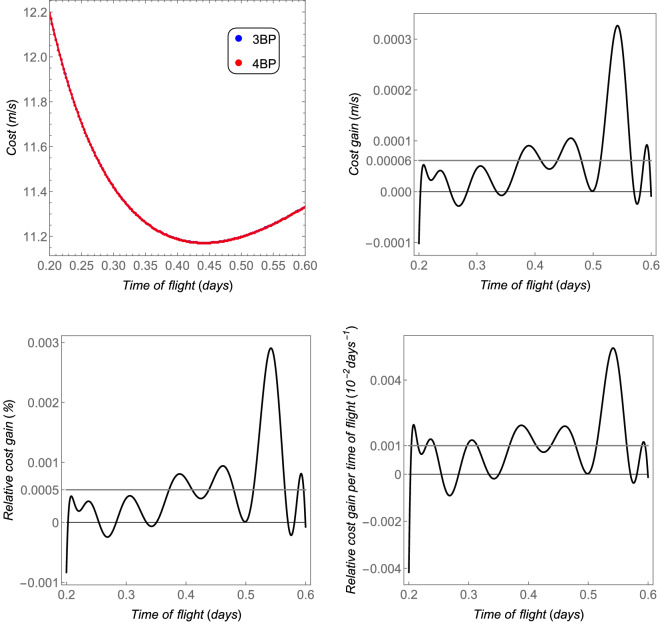
The total costs and the equivalent fuel savings of the 4BP in comparison with the 3BP for the pair Ida–Dactyl. The horizontal gray thicker straight lines are the average values.

The *cost*, *cost gain*, *relative cost gain*, and also the *relative cost gain per time of flight* are shown in Fig. 11, for a transfer from a circular orbit around Ida whose radius is 60 km, to another circular orbit around Dactyl whose radius is 2 km. Clearly, all indices show that both the 3BP and the 4BP coincide for the Ida–Dactyl system. As explained before, the closer the region is to the barycenter, the more the results for the 3BP coincide with the ones obtained from the 4BP. The average value of the *relative cost gain per time of flight* is only 0.001% per day, which is the lowest one among the cases shown in this section. Note that the numerical proceedings was done with higher precision for this case, in order to evaluate the very small differences (the average *relative cost gain* is only 0.0005%). Possibly, a more refined numerical evaluation could give an even smaller difference between the 3BP and the 4BP, at the cost of an increased computational cost. On the other side, the smallest *relative cost gain per time of flight* is in agreement with the smallest relative perturbation among the cases studied in this section.

### Comparisons of the results obtained for the Solar system

In this section, the results obtained in the previous sections for several pairs in the Solar system will be analyzed, based in the data obtained from the previous subsections shown in Table 5, where the averages of the indices defined in “Orbit transfer using a bi-impulsive maneuver” section are shown. The values of the *relative cost gain per time of flight* may be separated into two groups, whose values are of the same order of magnitude. The first one is given by the Sun–Earth–Moon and the Sun–Mars–Phobos systems and the second group is given by the Sun–Saturn–Titan and Sun–Ida–Dactyl systems. Although the gravitational parameters of Phobos is much smaller than the gravitational parameter of the Moon (by seven orders of magnitude), the distances involved in the travel (the parameter *R*) are also much smaller in the case of a travel between Mars and Phobos than the distances between the Earth and the Moon. This means that the spacecraft is much closer to the barycenter of the smaller primaries in a Mars to Phobos transfer than it is in the case of an Earth to Moon transfer. The final result is that the savings are similar for transfers in both systems. The comparison between the Sun–Saturn–Titan and the Sun–Ida–Dactyl can also be done using a similar analysis. On one side, the gravitational paramater of Titan is ten orders of magnitude greater than the gravitational parameter of Dactyl, and, on the other side, the parameter *R* is five orders of magnitude lower for the Sun–Ida–Dactyl in comparison with the Sun–Saturn–Titan system. This means that travels in the Ida–Dactyl system are made much closer to the barycenter compared to travels between Saturn and Titan. The final effect is that, for both systems, the influence of the perturbation due to the Sun over the fuel savings is negligible.

**Table 5:** Values of the parameters and the indices for several systems, where $AV_1$ is the average *cost gain*, $AV_2$ is the average *relative cost gain*, and $AV_3$ is the average *relative cost gain per time of flight*.

Parameters and indices	System			
	Sun–Earth–Moon	Sun–Mars–Phobos	Sun–Saturn–Titan	Sun–Ida–Dactyl
$\mu _e\,(\text {m}^3/\text {s}^2)$	$4.0\times 10^{14}$	$4.3 \times 10^{13}$	$3.8 \times 10^{16}$	$3\times 10^{6}$
$\mu _m\,(\text {m}^3/\text {s}^2)$	$4.9\times 10^{12}$	$7.2 \times 10^{5}$	$9.0 \times 10^{12}$	$2.7 \times 10^{2}$
$R\,(\text {m})$	$3.8\times 10^{8}$	$9.4\times 10^{6}$	$1.2 \times 10^9$	$9.1 \times 10^4$
$R_s\,(\text {AU})$	1.0	1.5	9.6	2.9
$AV_1\,(\text {m}/\text {s})$	2.17	0.57	1.29	0.00006
$AV_2\,(\%)$	0.055	0.031	0.013	0.0005
$AV_3\,(\%/\text {days})$	0.0118	0.013	0.003	0.001

### Variation of the parameters for the Solar system

In this section, the behavior of the relative perturbation $\Vert {{\varvec{p}}}_s\Vert /\Vert {{\varvec{p}}}_m\Vert$ is analyzed as a function of the parameters involved in the problem. Note that the relative perturbation is a function of *x*, *y*, *z*, and $\theta _2$. In order to simplify the analysis, two averages are performed. The first average is taken for the relative perturbation over a straight line from the position of $\mu _e$ $(-d_e,0,0)$ to the position of $\mu _m$ $(d_m,0,0)$, according to

18 $$\begin{aligned} \text {average}_1\bigg (\frac{\Vert {{\varvec{p}}}_s\Vert }{\Vert {{\varvec{p}}}_m\Vert }\bigg ) =\frac{1}{R}\bigg (\int _{-d_1}^{d_2}\frac{\Vert {{\varvec{p}}}_s\Vert (x,0,0,\theta _2)}{\Vert {{\varvec{p}}}_m\Vert (x,0,0)}\mathrm {d}x\bigg ) \end{aligned}$$

A second average is taken for the position of the Sun $\theta _2$ from 0 to $2\pi$, according to

19 $$\begin{aligned} \text {averages}\bigg (\frac{\Vert {{\varvec{p}}}_s\Vert }{\Vert {{\varvec{p}}}_m\Vert }\bigg ) =\frac{1}{2\pi }\bigg (\int _{0}^{2\pi }\text {average}_1 \bigg (\frac{\Vert {{\varvec{p}}}_s\Vert }{\Vert {{\varvec{p}}}_m\Vert }(\theta _2)\bigg )\mathrm {d}\theta _2\bigg ) \end{aligned}$$

Once these averages are taken, the dependency of the relative perturbation on the position on the space (*x*, *y*, *z*) and on the direction of the Sun $(\theta _2)$ are removed. The average on the position provides a good estimation of the order of magnitude of the relative perturbation. It was seen that the magnitude of the perturbation $\Vert {{\varvec{p}}}_s\Vert$ is a distorted sphere around the barycenter, thus, the average on the direction of the Sun $\theta _2$ can also provide a good estimation of its order of magnitude. Furthermore, the major interest here is to investigate the general behavior of the relative perturbation, not its local variation.

Assuming that $\mu _s$ is the gravitational parameter of the Sun, the averages are functions of four parameters: $\mu _e$, $\mu _m$, $R_s$, and *R*.

**Figure 12:**
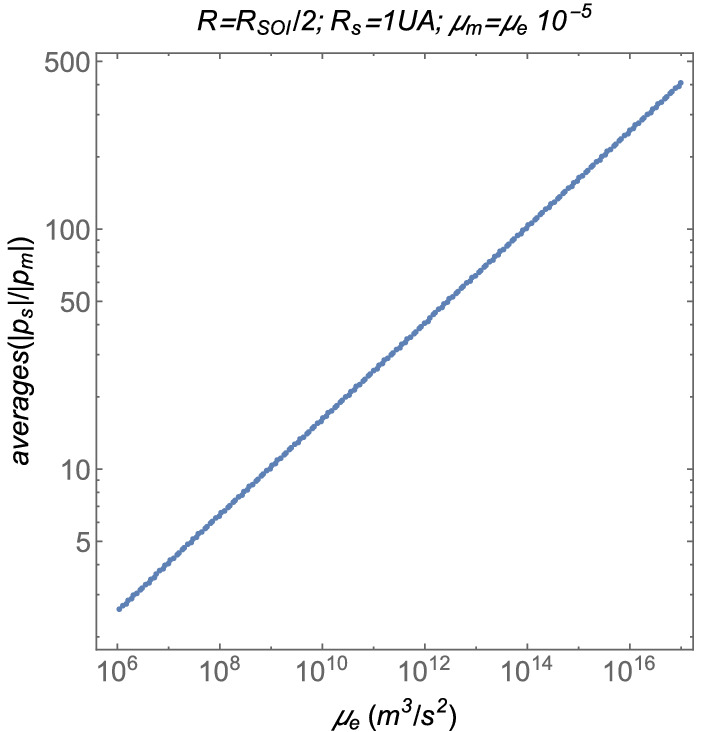
The averages as functions of the gravitation paramater $\mu _e$.

The first case to be analyzed is the dependency of the averages on the parameter $\mu _e$. Note that the parameter *R* is, in general, lower than the sphere of influence of $\mu _e$. Hence, for this case, the value of the parameter *R* is $R=R_{SOI}/2$, where the sphere of influence is $R_{SOI}=R_s\big (\frac{\mu _e}{\mu _s}\big )^\frac{2}{5}$, according to^39^. In this case, the gravitational parameter of the moon is $\mu _m=\mu _e\times 10^{-5}$. Finally, the distance from the Sun is $R_s=1~\text {AU}$. In this case, the averages as functions of $\mu _e$ are shown in Fig. 12.

**Figure 13:**
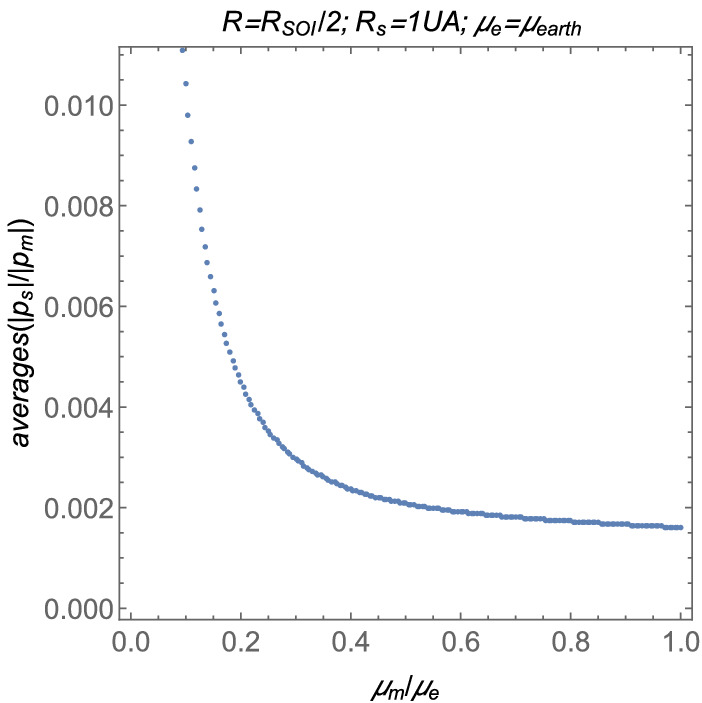
The averages as functions of the gravitation paramater $\mu _m$.

The second case to be analized here is the dependency of the averages on the parameter $\mu _m$. The other parameters are such that $R_s=1~\text {AU}$, $R=R_{SOI}/2$, and $\mu _e$ is the gravitational parameter of the Earth, whose value is shown in Table 1. In this case, the averages as functions of $\mu _m$ are shown in Fig. 13.

The variations of the averages with the parameter $R_s$ are also analyzed. In this case, the other parameters are such that $R=R_{SOI}/2$, $\mu _e$ is the gravitational parameter of the Earth, shown in Table 1, and $\mu _m=\mu _e\times 10^{-3}$. In this case, the evaluations showed that they have $\text {averages}\big (\frac{\Vert {{\varvec{p}}}_s\Vert }{\Vert {{\varvec{p}}}_m\Vert }\big )=1.42144$ for every value of $R_s$ in the range $10^{9}~\text {m}<R_s<10^{13}~\text {m}$.

**Figure 14:**
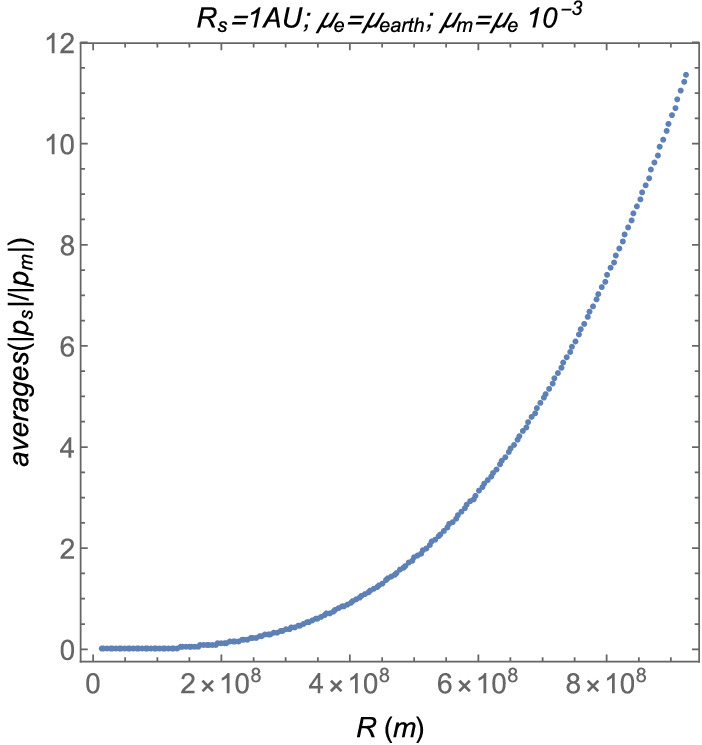
The averages as functions of the distance between the two smaller main bodies *R*.

The averages as functions of *R* are shown in Fig. 14. The other parameters are given by $R_s=1~\text {AU}$, $\mu _e$ is the gravitational parameter of the Earth, shown in Table 1, and $\mu _m=\mu _e\times 10^{-3}$.

## Conclusion

The present paper developed a new method to accurately measure the differences between the CR3BP and the bi-circular bi-planar 4BP dynamical models, which is particularly important in the regions of transfers around the smaller primaries. This method can be used to choose the model to be used in real mission design, evaluations of trajectories, optimization of computational time and fuel consumption, with high accuracy.

To develop this method, a term provided by the bi-circular bi-planar four body problem due to the influence of the Sun is added. It is seen as a perturbation, which explicit shows the differences between the bi-circular bi-planar four body problem and the CR3BP. The lower this term, the closer the bi-circular bi-planar four body problem is to the CR3BP.

Firstly, it was analytically shown (for any mass of each one of the three bodies) that the perturbation of the Sun is null at the barycenter of the system composed by $M_e$ and its moon. After that, it was also shown that the perturbation is linearly proportional to the position of the satellite with respect to the barycenter of the $M_e$-moon system, when the satellite is at a fixed distance $R_s$ from the Sun, which can be seen as a straight line transversal to the direction of the Sun, for a satellite close to the Earth. Finally, the numerical results confirmed the concentric oval curves for the same values of the magnitude of the perturbation, although it also shows that the direction of the perturbation have different symmetries.

In general, the results show that the closer the spacecraft is to the Earth–Moon barycenter, the lower the magnitude of the perturbation. It happens due to the inclusion of the Sun in the equations of motion, and, hence, the closer the bi-circular bi-planar four body problem is to the circular restricted three body problem.

Investigations were performed by evaluating orbit transfers in several pairs of bodies in the Solar system, like Earth–Moon, Mars–Phobos, Saturn–Titan, and the Ida–Dactyl systems. The results showed that the lowest perturbation of the Sun is for the Ida–Dactyl pair, which means that the CRTBP and the bi-planar bi-circular 4BP gives very similar results for this system. The system Saturn–Titan showed to have the second lowest differences between the two models. The third lowest differences were found for the Earth–Moon system. Finally, the larger differences were found for the Mars–Phobos system. It means that the CR3BP is not a good model to study this system. The influences of these costs are strictly linked to the fuel savings when using the bi-circular 4BP in comparison with the CR3BP, depending on the position of the Sun relative to the smaller primaries. These savings are evaluated and shown in this paper considering short time transfers.

Although the coincidence of the CR3BP and the bi-planar bi-circular 4BP depends on the combinations of the masses of the pair $M_e$ and its moon, its internal distance, and their distances from the Sun, it was found that the differences between the models increase with the masses of the pair and with their internal distance from the common barycenter.

This research also has the potential for a new approach to help to explain the tidal effects on the Earth due to the Sun, since the level of perturbation can also be measured in the surface of the Earth. Note that, since the Earth is displaced from its barycenter with the Moon (see Fig. 2), the perturbation of the Sun is not symmetric over the surface of the Earth. The magnitude of the perturbation is lower for the instantaneous side that is closer to its barycenter with the Moon and much larger in the opposite side, which generates the tidal effects on the Earth.
